# Synchronous HER2-Positive Breast and Gastric Cancers: A Dual Diagnostic Challenge With a Single Treatment Possibility

**DOI:** 10.7759/cureus.111402

**Published:** 2026-06-24

**Authors:** Jeremi Morka, Paula Biernat, Anna Czerwinska, Klaudia Cybulska, Dagmara Cybulska, Aleksandra Rysak, Michal Sobczak, Tomasz Mróz

**Affiliations:** 1 Faculty of Medicine, University of Opole, Opole, POL; 2 Medicine, University Clinical Hospital in Opole, Opole, POL; 3 Internal Medicine, University Clinical Hospital in Opole, Opole, POL; 4 Faculty of Medicine, Medical University of Silesia in Katowice, Katowice, POL

**Keywords:** breast cancer, erbb2 amplification, gastric cancer, her2-positive cancers, multiple primary cancers, synchronous cancers, targeted therapy, trastuzumab

## Abstract

Synchronous primary malignancies are rare and represent a significant diagnostic and therapeutic challenge, particularly when both tumors share a targetable molecular alteration. We present a case of synchronous human epidermal growth factor receptor 2 (HER2)-positive breast and gastric cancers treated using a common HER2-directed strategy. A 77-year-old female was admitted with a right breast lesion classified as Breast Imaging Reporting and Data System (BI-RADS) 5. A core needle biopsy was performed, which confirmed a grade 2 invasive ductal carcinoma. The results showed positivity for estrogen receptor and progesterone receptor, a HER2 immunohistochemical score of 2+, and a Ki-67 index of 15%. Chromogenic in situ hybridization (CISH) confirmed HER2 amplification, establishing a luminal B/HER2-positive subtype (cT4b cN0 cM0). The patient was started on a course of tamoxifen treatment. During the course of treatment, there was a progression of dysphagia and rapid weight loss, which prompted further investigation. A CT scan revealed thickening of the gastric cardia. Following the failure of gastroscopies due to esophageal stenosis, exploratory laparoscopy was performed. The histopathological examination revealed that the gastric cardia tumor was grade 1 tubular adenocarcinoma, with HER2 overexpression (immunohistochemistry (IHC) 3+), proficient mismatch repair (pMMR)/microsatellite stability (MSS) status, and no hormone receptor expression. Due to the unresectable nature of the disease, the patient received a combination of palliative mFOLFOX6 (leucovorin calcium (folinic acid), fluorouracil, and oxaliplatin) and trastuzumab, in addition to ongoing endocrine therapy. Following four cycles, imaging showed disease stabilization, with decreased cancer antigen 19-9 (CA 19-9) and carcinoembryonic antigen (CEA) levels, and evidence of local tumor regression. Despite an initial positive response, the patient subsequently experienced disease progression and clinical deterioration after three months. The overall survival rate was 11.25 months. This case demonstrates the importance of comprehensive molecular diagnostics and the potential of HER2-targeted therapy as a unified treatment approach for synchronous HER2-positive malignancies.

## Introduction

Synchronous cancers refer to cancers diagnosed simultaneously, whereas metachronous cancers refer to two cancers diagnosed independently that occur two to six months apart [1]. Research suggests that synchronous and metachronous cancers account for a small percentage of secondary cancer diagnoses. A 25-year retrospective study involving 109,054 patients found that 1.63% developed multiple primary cancers, with 70.87% of these cases occurring metachronously (after two months) and 29.13% synchronously (within two months) [2]. Another review reported that the frequency of multiple primary malignancies ranges from 2% to 17% [3]. Moreover, a 2014 cross-sectional study found that among patients diagnosed with primary breast cancer, 10% developed secondary malignancies, with 3% classified as synchronous and 7% as metachronous cancers [4]. However, identifying cases in the literature where both diagnosed cancers exhibit human epidermal growth factor receptor 2 (HER2) gene amplification remains challenging. In this particular case, the diagnosis enabled treatment of both cancers with a single drug, i.e., trastuzumab, a monoclonal antibody that selectively binds to the HER2 receptor.

## Case presentation

**Figure 1:**
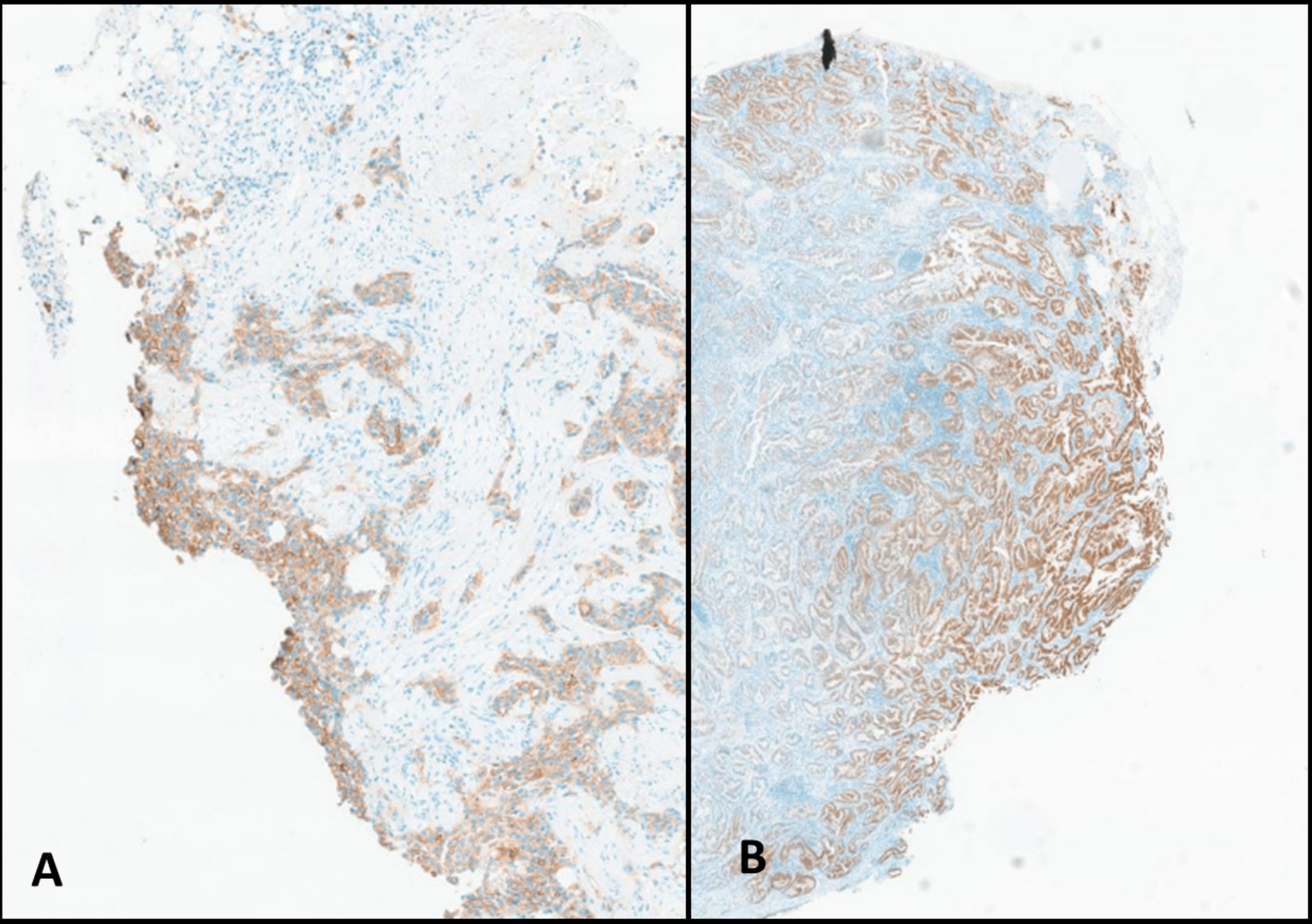
(A) Immunohistochemical staining for HER2 (score 2+) in invasive ductal carcinoma of the breast. Tumor cells demonstrate moderate, incomplete to circumferential membranous staining in a subset of neoplastic cells, corresponding to an equivocal HER2 expression pattern (IHC 2+). (B) Immunohistochemical staining for HER2 (score 3+) in well-differentiated (G1) tubular adenocarcinoma of the gastric cardia. Tumor cells exhibit strong, complete membranous staining in the majority of neoplastic cells, consistent with HER2 overexpression (IHC 3+). IHC: immunohistochemistry.

A 77-year-old female patient was admitted to the oncology department with the diagnosis of right breast tumor, Breast Imaging Reporting and Data System (BI-RADS) 5, detected on mammography. The diagnosis was confirmed by a core needle biopsy (CNB) and a histopathological examination, which led to a diagnosis of grade 2 infiltrating ductal carcinoma with expression of estrogen receptors (ER+), progesterone receptors (PR+), and HER2+, with an immunohistochemical score of 2+ (Figure 1A) and a Ki-67 proliferation index of 15%. Chromogenic in situ hybridization (CISH) confirmed HER2 gene amplification and established the biological subtype of the infiltrating carcinoma as luminal B, HER2-positive. At that stage, trastuzumab therapy could not yet be initiated because the immunohistochemical HER2 results were available only after a delay of several days. Consequently, the initiation of trastuzumab treatment coincided with the diagnosis of HER2 overexpression in the second primary malignancy, which was established seven days later. The disease was staged as IIIB (cT4b, cN0, cM0). At the beginning of the treatment, only a selective estrogen receptor modulator, specifically tamoxifen, was administered at the dose of 20 mg per day. The patient's increasing dysphagia and progressive weight loss (10 kg within 1.5 months) prompted the extension of the diagnosis. A computed tomography (CT) scan was performed and showed a thickening of the stomach wall in the area of the cardia. Two attempts to verify the diagnosis by gastroscopy were unsuccessful due to the difficulty of guiding the gastroscope into the stomach as a result of the esophageal stenosis. As a result of an undiagnostic gastroscopy, the patient was qualified for an exploratory laparoscopy. During the procedure, samples were obtained from the gastric tumor sections. Histopathological examination resulted in a diagnosis of a grade 1 tubular adenocarcinoma of the gastric cardia. Immunohistochemistry staining revealed HER2 protein in cancer cells with an immunohistochemical score of 3+ (Figure 1B), proficient mismatch repair (pMMR)/microsatellite stability (MSS), and no expression of the ER and PR. The tumor was considered unresectable due to complete infiltration of the visceral trunk (cT4b). Following a multidisciplinary team (MDT) discussion at the time of diagnosis, and taking into account the patient’s advanced age and significant comorbidities, surgical treatment was abandoned due to the high risk of catastrophic hemorrhage complications associated with resection of a tumor infiltrating the visceral trunk; consequently, the patient was deemed unsuitable for radical treatment and was qualified for palliative management. The principal therapeutic goal was to restore gastrointestinal continuity, thereby allowing the resumption of oral feeding. The patient was qualified for palliative immunochemotherapy according to the regimen of mFOLFOX6 (leucovorin calcium (folinic acid), fluorouracil, and oxaliplatin). Trastuzumab as a treatment for both cancers was also incorporated into the treatment plan. A three-week dosing regimen was used, consisting of a loading dose of 8 mg/kg body weight, followed by maintenance doses of 6 mg/kg administered every three weeks, starting three weeks after the loading dose. Previously implemented hormone therapy for breast cancer was also maintained. CT evaluation after four cycles of the treatment showed stabilization of the disease, and the evaluation showed a significant decrease in cancer antigen 19-9 (CA 19-9) and carcinoembryonic antigen (CEA) markers (Table 1) associated with local regression of gastrointestinal cancer. Treatment was maintained for three months; after that, a deterioration in the general condition of the patient and marker progression was noted. CT evaluation also showed progression. While consideration was being given to second-line treatment, the patient's condition rapidly deteriorated, leading to her death. Overall survival (OS) was 11.25 months.

**Table 1:** Serum tumor marker levels (CEA and CA 19-9) at diagnosis and after four cycles of chemotherapy. Both markers were elevated at baseline (CEA reference: <2.5 ng/mL in non-smokers, <5 ng/mL in smokers; CA 19-9: <37 U/mL) and showed a decrease after treatment, although values remained above the reference ranges. CEA: carcinoembryonic antigen; CA 19-9: cancer antigen 19-9.

Tumor marker	Baseline (at diagnosis)	After 4 cycles of chemotherapy	Change (%)
CEA (ng/mL)	21.5	14.6	-32.09%
CA 19-9 (U/mL)	565	321	-43.90%

## Discussion

According to the World Health Organization (WHO), in 2022, breast cancer was diagnosed in 2.3 million patients worldwide, leading to approximately 670,000 deaths. The disease was classified as the most prevalent cancer among adult women worldwide, with a high number of new cases observed in all age groups [5]. The Surveillance, Epidemiology, and End Results (SEER) database reports that the proportion of HER2-positive breast cancer is approximately 14% of the total, regardless of the expression of the hormone receptors [6].

HER2 is a transmembrane receptor tyrosine kinase in the epidermal growth factor receptor (EGFR) family, and its overexpression leads to constitutive activation of downstream signaling cascades, such as the phosphatidylinositol 3-kinase/protein kinase B (PI3K/AKT) and mitogen-activated protein kinase (MAPK) pathways, which promote unchecked cellular proliferation, survival, and metastasis [7]. In particular, HER2 forms heterodimers with other members of the ErbB family, notably HER3. This interaction activates both the PI3K/AKT and MAPK pathways, contributing to tumorigenesis by enhancing cellular proliferation and survival [8]. HER3, despite lacking kinase activity, is a critical partner for HER2, as it provides docking sites for key signaling molecules like PI3K and activates the downstream pathways involved in oncogenic processes. This HER2-HER3 heterodimerization is a central mechanism driving HER2-positive cancers and contributes to their aggressive nature [9].

The identification of HER2 as a therapeutic target has revolutionized the management of these malignancies, with the development of monoclonal antibodies such as trastuzumab and pertuzumab, alongside small-molecule inhibitors like lapatinib [10]. HER2 overexpression is confirmed using immunohistochemistry (IHC) or fluorescence/chromogenic in situ hybridization (FISH/CISH), with FISH being particularly recommended in cases of ambiguous IHC results [11].

HER2 receptor overexpression is associated with a poor prognosis, characterized by rapid tumor growth, an aggressive course, and frequent distant metastasis. Stocker et al. (2020) emphasized that the prognostic value of HER2 status is not only determined by the presence of amplification but also by the detection method employed. While IHC remains widely accessible, FISH is recommended when IHC results are ambiguous, particularly in cases of moderate or equivocal staining. Their study underlines the importance of accurate HER2 assessment to guide treatment decisions, as HER2-targeted therapies have notably improved outcomes for patients with HER2-positive breast cancer, underscoring the critical role of precise diagnostic techniques in managing this aggressive cancer subtype [12].

In 2020, gastric cancer was diagnosed in approximately 1.1 million patients globally, leading to 770,000 deaths [13]. The rate of HER2 receptor overexpression in gastric cancer varies widely, with studies reporting rates between 9% and 38%, depending on the research methodology and population studied [14]. Although second primary malignancies such as breast and gastric cancers are relatively common, the simultaneous presentation of HER2-dependent neoplasms in a single patient is unusual. Trastuzumab, a targeted therapy, has been used in the treatment of synchronous breast and colon cancers [15] as well as breast cancers combined with pathologically confirmed ipsilateral supraclavicular lymph node (ISCLN) metastases [16], highlighting the clinical challenge and importance of addressing multiple HER2-positive malignancies concurrently.

Tucatinib, a selective HER2 tyrosine kinase inhibitor, has demonstrated significant efficacy when combined with trastuzumab and capecitabine, especially in patients with HER2-positive metastatic breast cancer, including those with brain metastases. Preclinical studies by Olson et al. (2023) provide strong evidence that combining tucatinib with T-DM1 enhances antitumor activity through a synergistic mechanism that increases HER2 internalization and degradation. This combination not only enhances HER2 pathway inhibition but also improves tumor suppression and survival outcomes in xenograft models, including those resistant to T-DM1 monotherapy. Moreover, tucatinib’s ability to penetrate intracranial tumors supports its potential as an optimal tyrosine kinase inhibitor (TKI) partner for HER2-targeted therapies. These findings strongly advocate for further clinical investigation of tucatinib and T-DM1 combinations, particularly in patients with brain metastases, to expand treatment options and improve patient outcomes [17].

In the case of our patient, palliative immunochemotherapy using the mFOLFOX6 regimen combined with trastuzumab, while continuing previously initiated hormone therapy, was deemed appropriate. Initially, there was a positive response to treatment, with noticeable shrinkage of the breast tumor. However, within three months, the patient's performance status declined, and ultimately, the therapy was ineffective due to the late diagnosis of stage cT4b cN0 cM0 breast cancer and the diagnosis of unresectable stage cT4b gastric cancer, both contributing to overall treatment failure.

The future of oncology appears highly promising, particularly with the growing integration of molecularly targeted therapies. Advances in genomics and biotechnology are enabling the development of more personalized treatments, targeting specific molecular pathways that drive tumor growth. This precision approach helps minimize damage to healthy tissues and reduces the side effects commonly associated with conventional treatments [18].

Studies suggest that combining molecularly targeted therapies with immunotherapy offers considerable potential for improving cancer treatment. For instance, research has shown that combining antibody-drug conjugates with immunotherapy can enhance treatment effectiveness. In animal models of HER2-positive breast cancer, trastuzumab emtansine demonstrated synergistic effects when combined with antibodies targeting cytotoxic T-lymphocyte-associated antigen 4 (CTLA-4) and programmed death-ligand 1 (PD-L1), resulting in improved outcomes [19]. As the identification of new oncogenes and tumor-specific biomarkers continues to refine our treatment approach, targeted therapies are poised to revolutionize cancer care, tailoring treatments to individual tumor profiles and improving outcomes across various cancer types [20].

In summary, the convergence of molecularly targeted therapies, immunotherapy, personalized medicine, and advanced diagnostics is transforming oncology. This integrated approach offers the prospect of more effective and less toxic cancer treatments, moving toward a future where cancer is a more manageable and less daunting disease.

## Conclusions

The presence of multiple tumors is a major challenge in clinical practice. The development of modern molecularly targeted therapies in breast cancer would not have been possible without the identification of the ERBB2 protooncogene in the last century. Understanding its role in the development of other types of tumors has opened up new opportunities to use a drug with the same mechanism of action against two different, independent cancers. Such an approach could significantly reduce the side effects associated with the use of multiple, different cancer treatments. To make this treatment strategy a reality, it is essential that oncologists work closely with pathologists to ensure accurate histopathological diagnoses and incorporate the most appropriate and effective therapies into patient care.
